# Sepsis secondary to multifocal *Enterococcus faecium* infection: A case report

**DOI:** 10.1097/MD.0000000000019811

**Published:** 2020-07-02

**Authors:** Xiao-qing Huang, Jun-ke Qiu, Cai-hong Wang, Lei Pan, Jie-kun Xu, Xiao-hong Pan, Xiao-bo Ji, Min-jie Mao

**Affiliations:** Department of Tuberculosis Intensive Care Unit Tuberculosis Diagnosis and Treatment Center of Zhejiang Province, Hang Zhou Red Cross Hospital, Hang Zhou, China.

**Keywords:** *Enterococcus faecium*, immunocompromised host, sepsis, tuberculosis

## Abstract

**Introduction:**

Nosocomial *Enterococcus faecium* (*E faecium*) infections are common among immunocompromised patients; however, sepsis caused by *E faecium* is rarely encountered in the clinical setting.

**Patient concerns:**

A 69-year-old woman with a previous history of tuberculosis (TB), developed symptoms of recurrent fever, paroxysmal cough, and exertional dyspnea for over 2 months before she presented to the hospital.

**Diagnosis:**

The patient was initially misdiagnosed with recurrent TB, and did not respond to anti-TB therapy. Culture results of blood, endotracheal necrotic tissue, and urine confirmed a diagnosis of multifocal *E faecium* infection.

**Interventions:**

On definitive diagnosis, the patient received intensive antimicrobial combination treatment with linezolid, teicoplanin, caspofungin, and voriconazole on the basis of antimicrobial susceptibility results.

**Outcomes:**

After transient improvement, the patient's condition deteriorated due to secondary infections, and the patient died after discharge against medical advice.

**Conclusion:**

*E faecium* bacteremia may cause sepsis in immunocompromised patients, and has a high mortality rate. Careful pathogen detection and early initiation of treatment is crucial to good patient outcome.

## 1 Introduction

Normal commensal organisms of the intestinal tract, the *Enterococcus* spp. was long considered innocuous when compared with *Streptococcus pyogenes*, *Staphylococcus aureus*, and other virulent opportunistic microorganisms.^[1–3]^ Increasingly however, a number of studies have documented a relatively high prevalence of iatrogenic infections or bacteremia with *Enterococcus* spp. (especially *Enterococcus faecium*) as the causative microorganism in immunocompromised hosts.^[4–8]^ Enterococci have a great ability to acquire antimicrobial resistance through mutations and transfer of genetic material, with resultant drug resistance and a consequent high mortality rate among patients. Given the intrinsic resistance to cephalosporins and a reduced susceptibility to aminoglycosides in *Enterococcus* spp., the commonest first-line therapeutic regimens for enterococcal infection are ampicillin and a combination of ampicillin and gentamicin.^[9–11]^ Nonetheless, there have been increasing reports of nosocomial infections with vancomycin-resistant enterococci (VRE) woldwide.^[6,12]^ The 30-day mortality of enterococcal infection is up to 45% and over 25% in vancomycin-resistant and -susceptible strains, respectively.^[5]^

Enterococcal septicemia induces a severe inflammatory response, which can predispose patients to secondary bacterial infection, and this is associated with a high incidence of septic shock and multiorgan failure, which may contribute to the associated high mortality rate.^[13]^ Therefore, early identification of enterococcal infection and prevention of sepsis is a key challenge in clinical practice. Here, we present a case of sepsis caused by multifocal *E faecium* infection that was initially treated with TB as the differential diagnosis. Nonetheless, a progressive deterioration in patient condition was unsurmountable despite intensive treatment.

## 2 Case report

**Figure 1:**
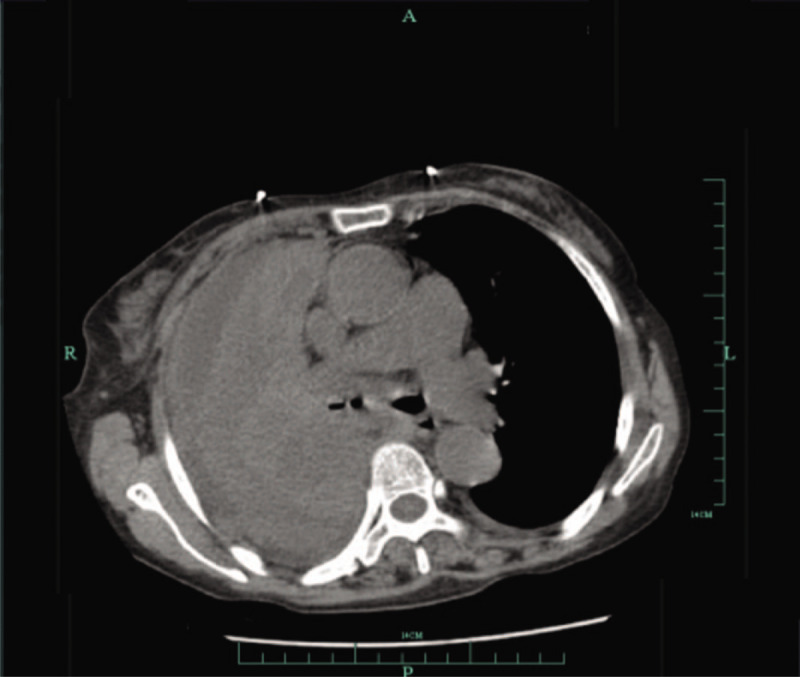
Chest CT scan showing right hilar soft-tissue mass associated with signs of congestive pneumonia and proliferative foci that had partial calcification in the right upper lobe.

**Figure 2:**
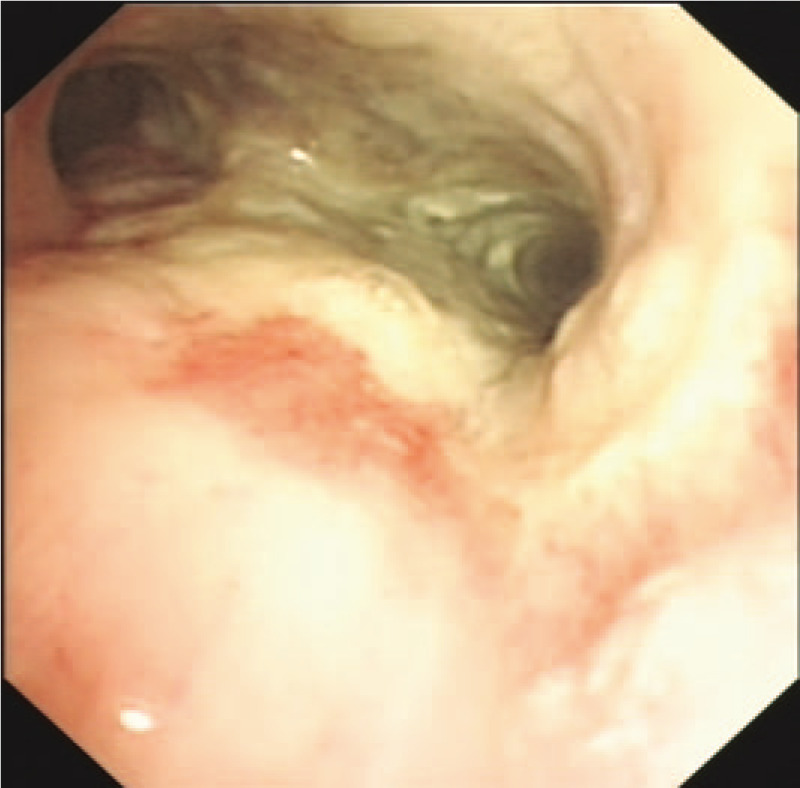
Bronchoscopy showing an obstruction of the right main bronchus by whitish necrotic tissue.

A 69-year-old female farmer, previously diagnosed and treated for tuberculosis (TB) over 20 years earlier with 6-month antitubercular treatment, presented with complaints of fever (maximum recorded temperature: 39 °C), paroxysmal dry cough, and exertional dyspnea since 2 months as well as hot flashes, sweating, and anorexia since 1 week. Although she was diagnosed with Sjogren syndrome 17 years back, she did not receive immunosuppressive treatment. The patient was initially hospitalized and treated at a local hospital, where she underwent a chest: computed tonograhy (CT) scan and bronchoscopy. The CT scan revealed a right hilar soft-tissue mass associated with signs of congestive pneumonia and proliferative foci that had partial calcification in the right upper lobe (Fig. 1). On bronchoscopy, the patient was found to have an obstruction of the right main bronchus by whitish necrotic tissue (Fig. 2), and biopsy specimens of the mass were obtained; histopathological examination of the biopsy specimens showed typical granulomatous changes, obvious caseous necrosis, and positive findings on acid-fast stain. Hematological and biochemical parameters were unremarkable at the time of her initial admission, but urine culture was positive for *E faecium*. The patient was diagnosed with secondary pulmonary tuberculosis, and she was started on the HREZS (isoniazid, rifampicin, ethambutol, pyrazinamide, and streptomycin) regimen. However, there was no response to treatment, and the patient was referred to the tuberculosis unit at our hospital.

On presentation at our hospital, the patient had persistent fever (38–40 °C) with increased airway secretions. The patient remained on antitubercular treatment (HREZS). The bronchoalveolar lavage fluid (BALF) culture showed the presence of *Candida tropicalis*, and antifungal therapy with an 8-day course of fluconazole was administered, followed by a 5-day course of voriconazole. Despite this treatment, the patient's condition deteriorated, and she was moved to the intensive care unit when she developed impaired consciousness, chest congestion and inability to expectorate, and blood oxygen desaturation. Following intubation and mechanical ventilation, blood oxygenation levels improved, and the patient regained consciousness. Laboratory findings include leukocytosis, neutrophilia, and high C-reactive protein (CRP) level, but the patient tested negative for TB on all TB-associated tests, including T-SPOT TB test, sputum smear test, sputum DNA/RNA test for mycobacterium tuberculosis and nontuberculous mycobacteria, histopathological examination of endotracheal necrotic tissue, GeneXpert test, and TB rapid culture with a BALF sample. In regard to the antitubercular treatment-refractive disease progression over 1 month, we excluded active TB and stopped antitubercular therapy. Results of blood culture and culture of the endotracheal necrotic tissue specimen were positive for *E faecium*, and the results of antimicrobial susceptibility tests are presented in Table 1. Throughput gene analysis of the BALF specimen detected *E faecium* (number of detected sequences 49,565). Therefore, the patient was diagnosed with sepsis secondary to multifocal *E faecium* infection, and combination therapy with meropenem (changed to linezolid after the antimicrobial susceptibility results), teicoplanin (instead of vancomycin because of renal insufficiency), caspofungin, and voriconazole was begun. We continued supportive care with mechanical ventilation, expectorant, and nutrient supplementation, and used noradrenaline vasopressor treatment for septic shock.

**Table 1:**
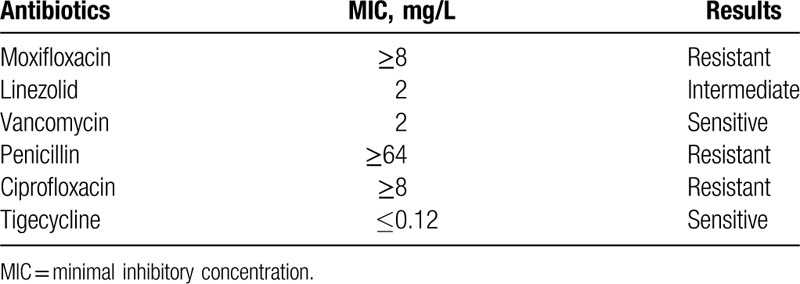
Antimicrobial susceptibility results for *E faecium.*

After 2 days of treatment, there was transient improvement in the general condition of the patient; body temperature, procalcitonin (PCT), and CRP levels showed a decreasing trend. Blood cultures turned negative, and WBC and neutrophil counts reverted to within the normal range. Subsequently, however, there was a recurrence of fever, dyspnea, productive cough, blood oxygen desaturation, and hypotension, and this necessitated a higher noradrenaline dosage as well as evaluation of the parameters of mechanical ventilation. Repeat sputum culture was positive for pan-drug-resistant *Klebsiella pneumoniae*, and the patient was placed on a modified antibiotic regimen with linezolid, teicoplanin, itraconazole, tienam, and tigecycline. Despite the treatment, a subsequent blood culture was positive for *Staphylococcus epidermidis*, and sputum culture was positive for *Burkholderia cepacian* and methicillin-resistant *S aureus* (MRSA); the patient's condition continued to deteriorate with the development of secondary infections, and the patient attendants requested for a discharge from the hospital against medical advice.

## 3 Discussion

There is a high prevalence of iatrogenic infection with multidrug-resistant *E faecium* among critically ill patients, especially the elderly, immunocompromised individuals, patients on long-term antibiotic therapy, ICU patients, and patients on mechanical ventilation. *E faecium* can cause opportunistic multisystem (e.g., urinary tract, digestive system, respiratory system, peritoneum, circulating system, and even nervous system) infections in immunocompromised individuals.^[5,14]^ Urinary and abdominal infections are the commonest source of enterococcal bacteremia.^[6,15]^ In the present case, the earliest evidence of *E faecium* infection was on urine culture; thus, the urinary tract was the likely source of bacteremia even in the absence of any urinary symptoms. However, gut-derived sepsis cannot be excluded because factors such as anoxia, stress response, and infection can impair the gut barrier and cause dissemination of intestinal flora.

In patients with hospital-acquired infection, especially in chronically hospitalized patients or those on multi-drug antibiotic treatment, the *Enterococcus* spp. is implicated as the second or third most commonly isolated pathogen. In individuals with infections that are not vancomycin-resistant, *E faecalis* and *E faecium* account for 85% to 90% and less than 10%, respectively, of the isolated Enterococcus.^[16,17]^ Among critically ill patients, such as those with a liver or stem-cell transplant, *E faecium* accounts for up to 40% of blood-isolated enterococcus. Moreover, *E faecium* accounts for 35% of enterococcus isolated from patients with nosocomial infections.^[18,19]^ Bacteremia is the most frequent manifestation of nosocomial *E faecium* infection, with the infection originating from the gastrointestinal tract, urinary tract, intravascular catheter, ulcerated or burned wounds, and so on. *E faecium*-associated endocarditis constitutes 5% to 15% of community-acquired and 30% of hospital-acquired endocarditis, which is usually confirmed on echocardiogram after a positive blood test. Despite the relative rarity of *E faecium*-associated cephalomeningitis, it can present in patients with a history of head injury, cranial surgery, or anatomical defects of the neural system. Thus, *E faecium*-associated cephalomeningitis is diagnosed on the basis of neurological symptoms, blood culture positivity for *E faecium*, and exclusion of other neurological abnormalities. The urinary tract is the most common site from which enterococci are detected, and urinary enterococcal infections can manifest as simple colonization, cystitis, pyelonephritis, perinephric abscess, prostatitis, and so on.^[20]^ However, enterococcal infections mostly develop only in the context of nosocomial infections, urological obstruction, catheterization, or an iatrogenic procedure.^[21,22]^*E faecium*, a less common enterococcus subtype, is more commonly detected in critically ill patients who have other underlying diseases. At our hospital, *E faecium* was the seventh commonest pathogen detected during 2015 to 2018, and 76% was detected from urine samples.

Severe pneumonia is the commonest pathological situation encountered in the ICU; most of the affected patients develop septic shock and respiratory failure, with a resultant high mortality rate, especially in elderly and immunocompromised individuals.^[23]^*S aureus* is a gram-positive coccus most frequently detected in the ICU, and MRSA strains account for 81.16% of these *S aureus* infections. *E faecium* is the second commonest pathogens, and 77.78% of these infections are caused by high-level aminoglycoside resistant (HLAR) strains. Nonetheless, *E faecium* bacteremia associated with pulmonary foci is rare, and early-stage diagnosis is challenging owing to a lack of typical manifestations.

Our patient had several predisposing factors that led to the development of *E faecium* pneumonia and bacteremia (post-pulmonary TB infection, immunocompromised status). The delay in identification and initiation of treatment for *E faecium* infection caused irreversible sepsis and subsequent secondary infection, and all of these resulted in patient mortality.

In addition, high drug resistance may be another reason for the poor patient outcome. *E faecium* exhibits strong adaptability to the environment and shows an intrinsic resistance to cephalosporins, aminoglycosides, trimethoprim/sulfamethoxazole, and clindamycin.^[24]^ Moreover, an increase in dosage or the number of antibiotics in the treatment regimen may further enhance the drug resistance of *E faecium*.^[15]^ A hospital-based survey of drug resistance in our hospital in 2017 (Table 2) showed *E faecium* was sensitive to only vancomycin, linezolid, and tigecycline, and these findings were consistent with the susceptibility results in the present case. The patient experienced transient improvement after administration of organism-sensitive antibiotics, which indicates the antibiotics were effective against the *E faecium* infection. However, the development of secondary infections caused irreversible deterioration of the patient's condition.

**Table 2:**
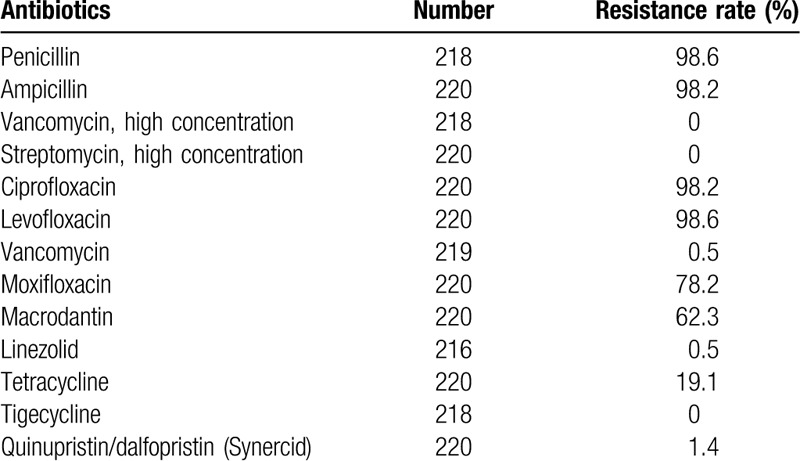
Drug-resistance profile of *E faecium* isolated at our hospital in 2017.

Rapid and accurate identification of bacterial pathogens is a key imperative in patients with sepsis or bacteremia to enable prompt initiation of appropriate therapy. In this patient, the isolation of *E faecium* in a urine culture was not accorded due attention at the beginning of her presentation. It is important for every positive result to be carefully interpreted and repeated over time. Use of some novel techniques, such as the genotype assay, can facilitate rapid detection of pathogens with reliable accuracy and specificity.^[25]^

In conclusion, sepsis caused by *E faecium* infection is a frequent iatrogenic infection, especially in immunocompromised individuals; however, its identification is challenging because of nonspecific manifestations. Rapid and accurate identification of *E faecium* infection and early initiation of the appropriate antibiotic treatment is the key to improving survival in the affected patients.

## Author contributions

**Conceptualization:** Xiao-qing Huang.

**Data curation:** Xiao-qing Huang, Jun-ke Qiu, Min-jie Mao.

**Formal analysis:** Xiao-qing Huang, Jun-ke Qiu, Xiao-bo Ji.

**Funding acquisition:** Jun-ke Qiu, Cai-hong Wang, Jie-kun Xu, Xiao-hong Pan, Xiao-bo Ji.

**Investigation:** Cai-hong Wang, Xiao-hong Pan.

**Methodology:** Xiao-qing Huang, Xiao-bo Ji.

**Project administration:** Xiao-qing Huang, Cai-hong Wang, Lei Pan.

**Resources:** Xiao-qing Huang, Lei Pan.

**Software:** Jie-kun Xu, Xiao-hong Pan.

**Supervision:** Lei Pan, Jie-kun Xu.

**Validation:** Min-jie Mao.

**Writing – original draft:** Xiao-qing Huang.

**Writing – review & editing:** Xiao-qing Huang.
